# Therapeutic Targeting of Pancreatic Cancer via EphA2 Dimeric Agonistic Agents

**DOI:** 10.3390/ph13050090

**Published:** 2020-05-10

**Authors:** Ahmed F. Salem, Luca Gambini, Parima Udompholkul, Carlo Baggio, Maurizio Pellecchia

**Affiliations:** Division of Biomedical Sciences, School of Medicine, University of California Riverside, 900 University Avenue, Riverside, CA 92521, USA; Ahmed.Farouk-Salem-Abdalla@medsch.ucr.edu (A.F.S.); lucaga@ucr.edu (L.G.); Parima.Udompholkul@medsch.ucr.edu (P.U.); Carlo.baggio@medsch.ucr.edu (C.B.)

**Keywords:** EphA2, agonistic EphA2 peptides, 135H12, cell migration, pancreatic cancer, drug discovery, targeted delivery

## Abstract

Recently, we reported on potent EphA2 targeting compounds and demonstrated that dimeric versions of such agents can exhibit remarkably increased agonistic activity in cellular assays compared to the monomers. Here we further characterize the activity of dimeric compounds at the structural, biochemical, and cellular level. In particular, we propose a structural model for the mechanism of receptor activation by dimeric agents and characterize the effect of most potent compounds in inducing EphA2 activation and degradation in a pancreatic cancer cell line. These cellular studies indicate that the pro-migratory effects induced by the receptor can be reversed in EphA2 knockout cells, by treatment with either a dimeric natural ligand (ephrinA1-Fc), or by our synthetic agonistic dimers. Based on these data we conclude that the proposed agents hold great potential as possible therapeutics in combination with standard of care, where these could help suppressing a major driver for cell migration and tumor metastases. Finally, we also found that, similar to ephrinA1-Fc, dimeric agents cause a sustained internalization of the EphA2 receptor, hence, with proper derivatizations, these could also be used to deliver chemotherapy selectively to pancreatic tumors.

## 1. Introduction

Pancreatic cancer is an extremely aggressive and deadly disease, which accounts for about 3% of all cancers in the United States and about 7% of all cancer deaths. It is estimated that in 2020 about 57,600 people (30,400 men and 27,200 women) will be diagnosed with pancreatic cancer and that 47,050 people (24,640 men and 22,410 women) will die of pancreatic cancer, (https://www.cancer.org/cancer/pancreatic-cancer/about/key-statistics.html). Unfortunately, most pancreatic cancers develop resistance to chemotherapy and radiation. Current therapeutic strategies include treatment with 4-drugs: fluorouracil, leucovorin, irinotecan and oxaliplatin [1], gemcitabine or, more recently, gemcitabine plus abraxane (nanoparticle albumin-bound paclitaxel) [2]. While these treatments have significant effect on patients’ overall survival, their therapeutic impact remains modest [3,4].

Unlike general chemotherapy, targeted therapies focus on attacking cancer-specific pathways that contribute to cell proliferations, suppression of apoptosis, or cell migration, all contributing to the aggressiveness of pancreatic cancer. One such emerging family of targets are the Eph receptors. Eph receptor tyrosine kinases are involved in a variety of cell–cell interactions, communicating via their ligands (the ephrins) [5,6,7,8,9,10,11,12,13,14,15,16,17,18,19,20]. In cancer, the unbound EphA2 subtype is pro-oncogenic, promoting angiogenesis and cell migration. In pancreatic cancer, EphA2 expression is dramatically inversely correlated with survival [9,21], and the detection of EphA2 fragments in plasma has been recently proposed as a new possible diagnostic approach to anticipate the aggressiveness of pancreatic cancer in patients [22]. More recent studies, underlined the role of EphA2 in driving therapy-resistant pancreatic adenocarcinomas, suggesting that EphA2 targeting agents should be developed and used in combination with current therapeutics [23]. In addition, our recent studies in a variety of pancreatic cancer cell lines, or primary pancreatic cancer tissues, revealed elevated EphA2 levels [24]. Activation of the receptor by its ligands (the ephrins) or by synthetic agonistic peptides, cause its internalization of the receptor, followed by its lysosomal degradation [25]. Hence agonistic peptides could be used to reduce EphA2 levels or, when properly conjugated with cytotoxic agents, could serve as peptide–drug conjugates to deliver chemotherapy to EphA2 expressing tumors [24,25,26,27,28,29]. For example, we previously demonstrated that earlier EphA2 targeting agonistic agents conjugated with gemcitabine had superior efficacy compared to gemcitabine alone in mice models of pancreatic cancer [24]. We subsequently demonstrated that dimeric versions of these agents possessed dramatically increased cellular efficacy in causing receptor activation, internalization, and degradation [26]. Very recently, we developed novel and more potent synthetic agents, which target the EphA2-LBD (ligand binding domain) at nanomolar concentrations, as corroborated by robust biophysical methods, including X-ray crystallography, and isothermal titration calorimetry (ITC), and biochemical data [30]. Here we focused on further investigating dimeric versions of these agonistic agents in pancreatic cancer cells, compared to a dimeric natural ligand (ephrinA1-Fc) and to EphA2 knockout cells.

## 2. Results and Discussion

### 2.1. Synthesis and Characterization of Dimeric and Tetrameric 12-Mers Targeting the EphA2-LBD

Recently, we reported that dimerization of EphA2 binding 12-mer peptides resulted in agents with dramatically increased agonistic activity in cell, presumably by catalyzing receptor dimerization and subsequent clustering [26,30]. In an attempt to further investigate the basis for this increased activity we prepared a variety of 12 mers and related dimeric and tetrameric agents as reported in Table 1. The synthesis of all agents followed the general solid phase strategies as we recently reported [30]. Dimeric agents were obtained likewise by a solid-phase synthetic scheme that introduced an additional Lys residue as the terminal amino acid, which allowed coupling of the C-terminus of each of two monomers onto its backbone and side chain amines, respectively (Table 1). Similarly, we also prepared a tetrameric agent by introducing an additional Lys-Lys di-peptide at the C-terminus of a dimer, which allowed two dimers to be conjugated into a tetramer (Table 1). To further investigate the effect of the linker length on the activity of the resulting dimers, we also introduced Gly, β-Ala, or γ-aminobutyric acid (GABA) at the C-terminus of the 12-mers, hence, prior to the terminal Lys residue used for the dimerization (Table 1). To characterize the binding properties of these novel agents, we adopted our recently developed dissociation-enhanced lanthanide fluorescent immunoassay (DELFIA) [30], where a biotinylated EphA2 binding peptide (123B9, Table 1) was prepared and used as bait in streptavidin-coated 96-well plates. Subsequently, recombinant 6xHis-EphA2-LBD and fluorescent europium-conjugated anti-6xHis antibody were added to each well. After a brief incubation time of the complex and a given test agent, followed by washing steps, residual fluorescence was measured. Dose response measurements were subsequently carried out to assess the ability of any given test compound to displace 123B9 from EphA2-LBD [30]. We showed that this assay is highly reproducible and produces data that align very well with both previously reported ELISA-based IC_50_ values, and isothermal titration calorimetry binding data [30]. Hence, using the DELFIA assay, IC_50_ values for each agent were obtained by dose-response displacement measurements and reported in Table 1.

Our previous optimizations studies started by analyzing the binding properties and sequences of YSA and ephrins-derived peptides [30]. These studies culminated with lead agent 135B12 (Table 1), which presented a significantly increased affinity for the receptor compared to YSA. Based on our previous experience with dimeric agents [26], we subsequently derived dimeric versions of 135B12 with various linker lengths (agents 135C11, 135C12, and 135D1) [30]. We also previously found that the stability of these peptides in plasma is limited, mostly because of aminopeptidases that can efficiently cleave the first amino acid [28]. However, we could dramatically improve plasma stability of these agents by replacing the N-terminal Tyr residue with bioisosters, such as the 2-(3-chloro-4-fluorophenoxy)acetic acid (included in agents 123B9 [27], and 135E2 [30]). We subsequently solved the X-ray structure of 135E2 in complex with EphA2-LBD [30] that allowed us to fine tune the composition of some of the side chains of this agent, leading first to agent 135G3 (and its dimer version 135G4) (Table 1), and subsequently to agent 135H11 in which the N-terminal residue was further optimized into a 3-CH_3_,6,7-OCH_3_,benzofuranoic acid (Table 1) [30]. Agents 135H12 and 135I1 are, respectively, the dimer and the tetramer version of 135H11 (Table 1). We did not expect that dimers would display dramatically increased affinities compared with their monomers for the isolated EphA2-LBD [26,30]. Nonetheless, some noticeable increased affinities (decreased IC_50_ values) were observed for some dimers and for the tetramer (Table 1). However, because we are interested ultimately in their ability to act as agonistic agents, we deferred rank ordering these multimeric agents to cell-based assays as reported in the sections below.

### 2.2. Dimeric Agents May Promote Dimerization of EphA2-LBD

**Figure 1:**
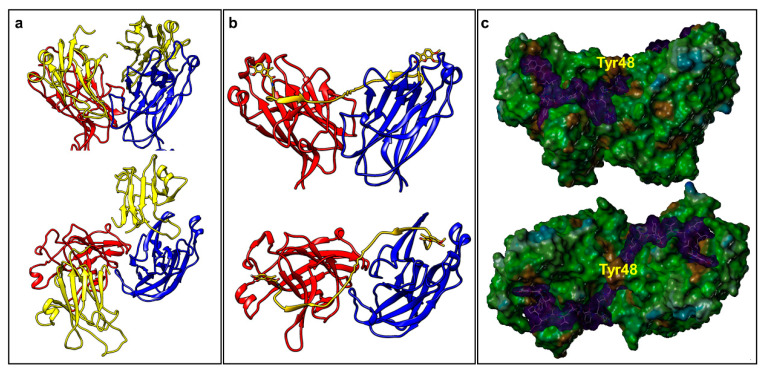
Molecular models of EphA2-LBD in complex with its agonistic agents. (**a**) Front view and top view of the molecular models representing the biological dimeric unit of EphA2-LBD (red and blue), in complex with ephrinA5 (yellow) (PDB ID 3MX0). (**b**) Front view and top view of the molecular model of 135H12 (yellow; Table 1) in complex with EphA2-LBD (red and blue). The model was build based on the dimeric biological unit of EphA2-LBD in complex with 135E2 (Table 1; PDB ID 6B9L). (**c**) Molecular surface representation of the models shown in (**b**) generated with MOLCAD (Sybyl-X 1.2). 135H12 surface is in magenta, while the surface for EphA2-LBD dimer is color coded according to hydrophobicity (MOLCAD). The position of residue Tyr48, at the bridge between the two monomer, is highlighted.

Our attempts to co-crystallize dimeric agents with EphA2-LBD have thus far been unsuccessful. However, we were recently able to derive for the first time the X-ray structure of an agonistic peptide agent (135E2, Table 1) in complex with EphA2-LBD (PDB ID 6B9L) [30]. The structure of ephrinA5 bound to EphA2-ectodomain has also been reported (PDB ID 3MX0) [31] (Figure 1a). Interestingly, the dimeric biological unit of 135E2 in complex with EphA2-LBD is nearly identical to the same dimeric arrangement found in the structure of the EphA2 ectodomain in complex with ephrinA1 (Figure 1). Hence, by simply fixing the geometry of this dimer and that of the crystallographic bound conformation of 135E2, we modeled dimeric 135H12 (Table 1) into the monomers (Figure 1b). After building the structure, the complex was energy-minimized, resulting in a minimal rearrangement of the receptor side chains and of the bound peptide with respect to the original experimental crystallographic structure. Interestingly, in this model the linker between two of the 12-mers is threaded through a flat narrow channel created by the interface between the two EphA2 monomers, and capped by EphA2 residue Tyr48 (one from each monomer, Figure 1c). In this model, similar to the dimer observed within the complex with ephrinA1 (Figure 1a), there seem to be only relatively limited contacts between the monomers. Interestingly, the space occupied by the linker is similar to the space occupied by a biotin moiety as recently reported in using a biotinylated YSA [32] (Table 1) peptide, that resulted more potent than the non-biotinylated agent [33]. 

**Figure 2:**
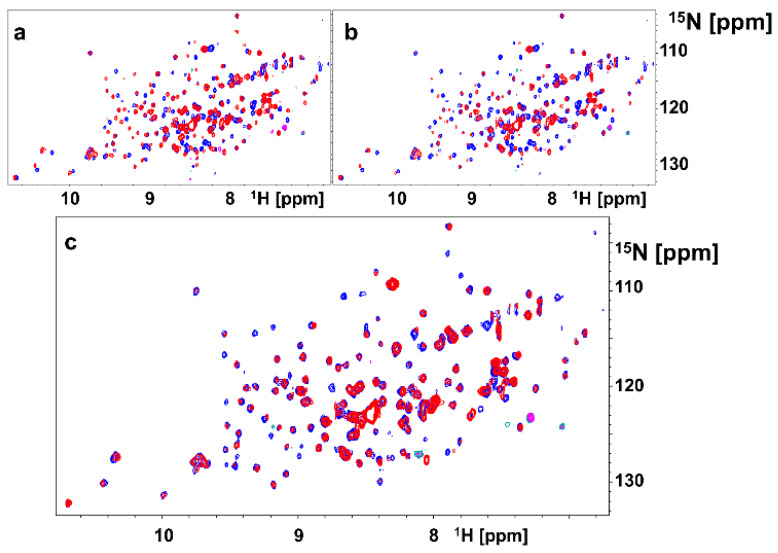
2D NMR [^15^N,^1^H] spectra with ^15^N-labeled EphA2-LBD. (**a**) Superposition of 2D NMR spectra of EphA2-LBD (20 μM), recorded in absence (blue) and presence (red) of 135G3 (20 μM). (**b**) Superposition of 2D NMR spectra of EphA2-LBD (20 μM), recorded in absence (blue) and presence (red) of 135G4. (**c**) Superposition of 2D NMR spectra of EphA2-LBD (20 μM), recorded in presence of 135G3 (blue) or in presence of 135G4 (red).

As mentioned above, our attempts to crystallize dimeric agents in complex with EphA2-LBD have thus far failed. However, we could express and purify ^15^N-labeled EphA2-LBD and have conducted comparative NMR experiments with monomers and dimers (Figure 2). 2D [^15^N,^1^H]-sofast HSQC NMR experiments were performed in presence and absence of stoichiometric amounts of monomer 135G3 or its dimer 135G4 (Table 1). Large changes in chemical shifts for several amide proton and nitrogen resonances were observed in the 2D [^15^N,^1^H]-sofast HSQC for each complex (Figure 2a,b), typical of potent and specific binding, as we recently reported [30]. However, comparison of the spectra of each complex revealed a differential line broadening of several resonances in the complex with 135G4 (dimer) versus the complex with 135G3 (monomer) (Figure 2c). These changes suggest that either chemical exchange (for example from monomer to dimer) and/or increased nuclear spin relaxation due to dimer formation and slower rotational correlation times of the complex are taking place, given that both events could contribute to the observed line broadening. While the line broadening is widespread, some resonances seem more affected and presented differences in chemical shifts. Unfortunately, the resonance assignments for EphA2-LBD are not available, but we speculate that these localized changes may reflect the formation, perhaps transiently, of the dimer interface. Hence, our modeling studies, based on the experimental structures of the monomers, and NMR data comparing monomers versus dimers binding suggests that, at the least transiently, the dimeric agents can favor dimer formation that could explain the dramatically increased agonistic activity in cellular assays of the dimers versus the monomers as illustrated below.

### 2.3. Dimeric and Tetrameric Compounds Are Potent Agonists of EphA2 Signaling

**Figure 3:**
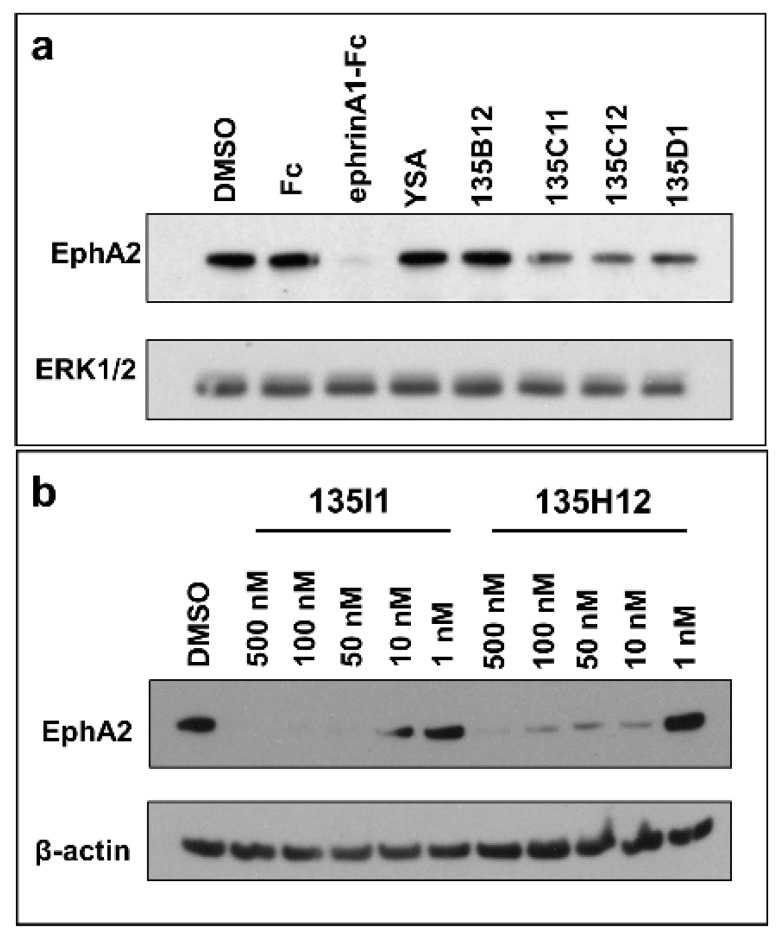
Dimeric and tetrameric EphA2 agonistic ligands degrade EphA2 receptor. (**a**) Western blot study of HCT116 cells treated with ephrinA1-Fc, EphA2 agonistic monomers (YSA, 135B12), and EphA2 agonistic dimers (135C11, 135C12, and 135D1) for 2.5 h (10 μM each). Total anti-EphA2 blot indicates that ephrinA1-Fc treatment led to complete degradation of the receptor, while EphA2 dimeric ligands treatments showed partial decrease of the receptor. Total ERK1/2 blot was used as a loading control. (**b**) Pancreatic cancer BxPC3 cells were treated with tetrameric (135I1) and dimeric (135H12) compounds for 1 h. These compounds successfully degraded EphA2 receptor at nanomolar concentrations.

In order to evaluate the ability of each agent to activate the receptor, we tested them in various cellular assays as reported below. Because receptor activation causes its internalization and degradation, we opted to monitor the ability of each agent to reduce EphA2 levels over time, after exposure of cells to test ligands. As control we used dimeric ephrinA1-Fc, as it was reported that monomeric ephrinA1 is less effective as an agonist compared to the Fc dimerized molecule [9]. First, we wanted to explore the effect of the linker length between the monomers and therefore tested side by side monomeric agent 135B12 and its dimeric versions 135C11, 135C12, and 135D1 (Table 1). The dimers introduced a Gly (135C11), a β-Ala (135C12), or a γ-amino butyric acid (GABA; 135D1) between the 12-mer (135B12) and the C-terminal Lys residue used to link the two monomers (Table 1). As controls we used ephrinA1-Fc, DMSO, YSA (an earlier agonistic peptide; Table 1) [32], and Fc. For these experiments we initially treated HCT116 cells with 10 μM concentration of each agent for 2.5 h, and cells lysates were probed for total EphA2 using anti-EphA2 antibody (1C11A12; Thermo Fisher Scientific) (Figure 3a). From this experiment, it seemed obvious that ephrinA1-Fc (at 1 μg/mL concentration) was very effective in causing EphA2 degradation, compared to controls (DMSO and Fc) and to monomeric agents YSA and 135B12 (Figure 3a). This is well in agreement with several previous studies with monomeric agents, including 135H11, that showed receptor activation only at relatively high concentrations (100 μM or higher) [26,30,32]. However, and in striking contrast, the dimeric versions of 135B12 exhibited huge reduction of EphA2 levels at this concentration for all dimers tested (Figure 3a). Given that no difference was observed between the dimers in inducing EphA2 degradation, we opted to select the dimer with the shortest linker (135C11) for further studies. Our linker length is also comparable with an earlier study with a less potent agonistic peptide of sequence SWLAYPGAVSYR that when dimerized at the C-terminus by an aminoexanoic acid linker resulted much more potent than its monomer in activating the receptor in cell [34]. Hence, using optimized agent 135H11, we then examined the ability of its dimeric (135H12) and tetrameric versions (135I1) (Table 1) to induce EphA2 degradation in the pancreatic cancer cell line BxPC3 (CRL-1687) (Figure 3b). Because these agents had optimized side chains [30], we expected these to work at much lower concentrations compared to dimeric agents derived from 135B12 (Table 1). Accordingly, test agents showed a remarkable induction of EphA2 degradation at nearly all concentrations tested (Figure 3b). Interestingly, the tetramer 135I1, despite it was significantly more potent in the DELFIA assay (Table 1), was not significantly more effective than its dimeric counterpart (135H12) in inducing receptor activation/degradation.

These data, all in all, support the conclusion that dimeric agents may facilitate dimer formation and subsequent receptor clustering and activation compared to monomeric agents, in agreement to our model and data reported in Figure 1 and Figure 2. Further, the data identified dimeric agent 135H12 as a promising agonistic compound of this series.

### 2.4. Cell Migration Studies

**Figure 4:**
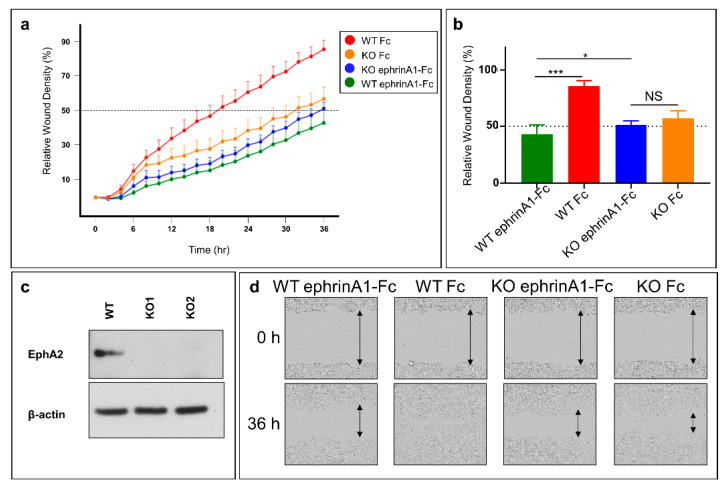
Genetic inhibition of EphA2 expression impairs migration of pancreatic cancer cells. (**a**) Real-time cell migration assay. WT and EphA2-KO BxPC3 cells were scratched and treated with either ephrinA1-Fc or Fc. Cells were left to heal for 36 h inside the IncuCyte S3 live-cell imager. WT cells treated with Fc migrated significantly faster than WT cells treated with ephrinA1-Fc (1 μg/mL). Moreover, knocking out EphA2 significantly reduced cell migration. However, treating EphA2-KO cells with ephrinA1-Fc did not have a significant additive effect on reducing cell migration. (**b**) Histogram of relative wound density at 36 h. (**c**) Validation of knocking out EphA2 using CRISPR-Cas9. WT and KO1 cell lines were used for the scratch assay. (**d**) Representative images of different scratches at times 0 and 36 h. *, *p* < 0.05; ***, *p* < 0.0001. Error bars represent standard deviation.

The monomeric, unbound EphA2 receptor is known to be pro-oncogenic, inducing cell migration of cancer cells, while ephrinA1-Fc, inducing EphA2 dimer formation can suppressed this activity [35]. To more directly examine the effect EphA2 on pancreatic cancer cell migration we first prepared a stable BxPC3 EphA2 knockout (KO) cell line and monitored its migratory properties using the scratch wound method and live-cell analysis (IncuCyte S3, Sartorius) (Figure 4). Briefly, homogeneous scratch wounds on plated cells were created by a 96-pin mechanical device (WoundMaker, Sartorius, Göttingen, Germany). Subsequently, live-cell imaging was performed to monitor the rate of wound closure. As controls, we also tested wild-type (WT) BxPC3 cells, and each cell type was treated with ephrinA1-Fc, or Fc as control (Figure 4). Cell migration was significantly attenuated in the EphA2-KO cells compared to WT-BxPC3 cells. In addition, the migratory properties of EphA2-KO cells was similar (not significantly different) to that of wild-type cells treated with ephrinA1-Fc (Figure 4a,b). Furthermore, treatment of EphA2-KO cells with ephrinA1-Fc did not significantly decrease the rate of migration (Figure 4). EphrinA1-Fc is a promiscuous ligand and could in principle activate several EphA or EphB receptor subtypes [36], hence any inhibitory effects observed when treating cells with ephrinA1-Fc may be due to a variety of Eph subtypes. However, the data reported in Figure 4 suggests that in BxPC3 the A2 subtype alone contributes to the pro-migratory properties of the cell line. 

**Figure 5:**
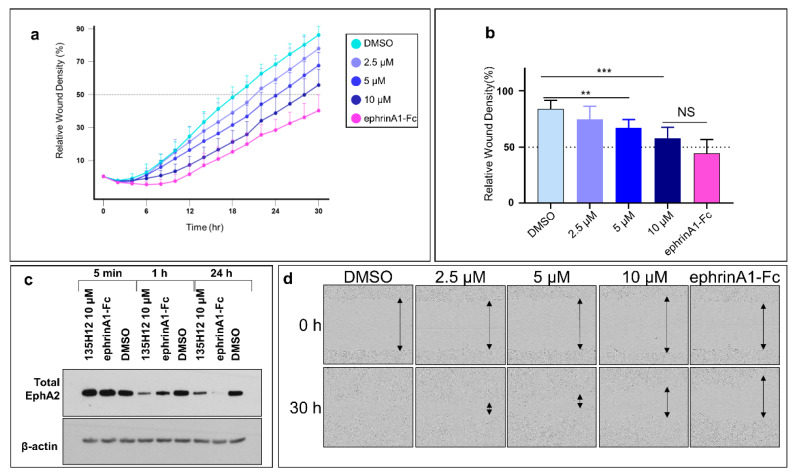
Pharmacological inhibition of EphA2 expression partially impairs migration of pancreatic cancer cells. (**a**) Scratched confluent WT-BxPC3 cells were treated with either 135H12 at different concentrations or 1 µg/mL ephrinA1-Fc and were imaged every 2 h. (**b**) Histogram of relative wound density after 30 h shows significant decrease of cell migration when treated with 5 µM and 10 µM of 135H12 or ephrinA1-Fc. (**c**) Western blot of BxPC3 cell lysates after exposure for 5 min, 1 h or 24 h to indicated agents. (**d**) Representative images from treated wells at times 0 and 30 h. **, *p* < 0.005; ***, *p* < 0.001. Error bars represent standard deviation.

Subsequently, we probed the effect of the pharmacological inhibition of EphA2 by our dimeric agents on the migratory properties of WT-BxPC3 in a similar assay (Figure 5a). The data revealed that the dimeric agent suppressed cell migration in a dose-response manner (Figure 5), at the highest concentrations tested (10 μM) its inhibitory effect on cell migration was similar (not significantly different) to that induced by ephrinA1-Fc treatment (Figure 5). Of note is that cell proliferation was not affected by either treatment with ephrinA1-Fc or by our agents.

## 3. Materials and Methods

### 3.1. Synthetic Chemistry

Fmoc (Fluorenylmethyloxycarbonyl)-amino acids, resins for solid synthesis and N-capping acids were obtained from commercial sources and used without further purification. Reported agents were synthesized in house by standard microwave-assisted Fmoc peptide synthesis protocols on Rink amide resin using a Liberty Blue Peptide Synthesizer (CEM Corp., Matthews, NC, USA). Briefly, typical reaction conditions included six equivalents of Fmoc-AA, three equivalents of DIC, and one equivalents of OximaPure in 4.5 mL of DMF (dimethylformamide). Each coupling reaction was conducted at 90 °C for 5 min in the microwave reactor, under constant nitrogen bubbling. Fmoc deprotection was performed by treating the resin-bound peptide with 20% piperidine in DMF (2 × 3 mL) for 3 min at 90 °C. Peptides were cleaved from the resin with a cleavage cocktail containing TFA(trifluoroacetic acid)/TIS/water/phenol (94:2:2:2) for 3 h, and the cleaved peptides were precipitated in cold Et_2_O, centrifuged and dissolved in DMSO. DMSO crude solutions were purified to >95% purity by preparative RP-HPLC using a Luna C18 column (Phenomenex, Torrance, CA, USA) on a JASCO preparative HPLC system and water/acetonitrile gradient (5% to 70%) containing 0.1% TFA. HRMS (high-resolution mass spectrometry) was used to assess the identity of the compounds. To prepare multimers, the amount of resin employed for the typical synthesis of monomeric 12-mers (0.1 mmol), was reduced to 0.05 (dimers) or 0.025 mmol (tetramer). An Fmoc-Lys(Fmoc)-OH was used at the branching points in the sequence. Double coupling was performed to ensure the complete reaction of all elongating sequences. Standard cleavage and purification protocol were used to obtain the pure dendrimers (purity >95% by HPLC).

### 3.2. Molecular Modeling and In Vitro Studies

Molecular modeling studies were conducted using Sybyl-X 1.2 (Certara, St. Louis, MO, USA) and the X-ray structures of 135E2 in complex with EphA2-LBD (PDB ID 6B9L) [30] and of ephrinA1 in complex with the ectodomain of EphA2 (PDB ID 3MX0) [31]. To prepare a model of 135H12 in complex with EphA2, the biological unit of the 135E2-EphA2-LDB complex was used and the side chains of 135E2 were first modified step-wise using the biopolymer routine of Sybyl, to obtain 135H11, and the resulting complex was energy-minimized. Subsequently, a C-terminal Gly-Lys linker was modeled in an extended conformation to 135H11, and covalently linked to a second 135H11 from the other unit of the biological unit dimer. The complex was further energy minimized (partial charges were assigned using the Gasteiger-Huckel method). Structural comparisons and molecular surfaces were obtained using Chimera (http://www.cgl.ucsf.edu/chimera) and MOLCAD, respectively (SYBYL-X 1.2, Ceratara, St. Louis, MO, USA). NMR studies were conducted on a ^15^N-labeled sample of EphA2-LBD that was obtained as described previously [25,27,30]. 2D so-fast-[^15^N,^1^H] HSQCs were measured on a 700 MHz Avance Bruker instrument equipped with a cryo-probe and automated sample changer. IC_50_ values reported in Table 1 were obtained via DELFIA (Dissociation-Enhanced Lanthanide Fluorescent Immunoassay) displacement assay as we recently described [30].

### 3.3. Cell Lines, Cell Culture, and Antibodies

BxPC-3 and HCT116, HEK273T/17 cell lines were purchased from the American Type Culture Collection (ATCC). All culture media and supplements were purchased from ThermoFisher and media were supplemented with 10% FBS and 1% Pen Strep to be completed. BxPC3 was cultured in complete RPMI-1640, and HCT116 and HEK293 were cultured in complete DMEM (Dulbecco’s Modified Eagle Medium). Anti-EphA2 antibody (1C11A12) was purchased from ThermoFisher, Waltham, MA, USA and anti-ERK1/2 antibody was purchased from Cell Signaling Technology, Danvers, MA, USA. β-Actin antibody was purchased from Santa Cruz Biotechnology, Dallas, TX, USA.

### 3.4. Establishment of an EphA2 Knocked-Out Pancreatic Cancer Cell Line

Human embryonic kidney HEK293T/17 cells were transfected with EphA2 CRISPR Guide RNA 1 plasmid (KO1; target sequence, CTACAATGTGCGCCGCACCG), or EphA2 CRISPR Guide RNA 2 plasmid (KO2; target sequence, AGGCTCCGAGTAGCGCACAC) which were purchased from GenScript and Expression Packing Kit (GeneCopoeia, Inc., Rockville, MD, USA) to produce lentivirus particles according to GeneCopoeia Inc’s protocol. After 2 days, viral particles were collected and filtered. Stable BxPC3 EphA2-KO cell lines were established by transducing with viral particles and selecting with 1 μg/mL puromycin 2 days post-transduction. EphA2-KO was confirmed by Western blot.

### 3.5. Cell Migration Assays

Cells were plated at 50 × 10^3^ cells/well density in 96-well ImageLock plates (Sartorius). The following day, cells were scratched using the WoundMaker (Sartorius) and washed three times with PBS. Subsequently, cells were treated with the indicated compounds in RPMI-1640 complete media. Treatments included ephrinA1-Fc (1 μg/mL) and test agents 135H12 (2.5 μM, 5 μM, and 10 μM). Plates were imaged every two hours using IncuCyte S3 (Sartorius), and relative wound areas were analyzed using the algorithm of the imager cell migration software module.

### 3.6. Immunoblotting Assays

Cells were lysed with cell lysis buffer (20 mM Tris, pH 7.4, 120 mM NaCl, 1% Triton X-100, 0.5% sodium deoxycholate, 0.1% SDS, 1% IGEPAL, 5 mM EDTA, supplemented with EDTA-free Protease Inhibitor Cocktail and PhosStop from Sigma-Aldrich, St. Louis, MO, USA) for 10 min on ice. Cell lysates were then centrifuged to clear off cell debris for 10 min at 13,000 rpm at 4 °C. Samples were prepared and loaded into 4–12% NuPAGE Bis-Tris Precast Gels and transferred to PVDF membrane as indicated previously [30]. The membrane was blocked with 5% non-fat milk in TBS and 0.1% Tween (TBST) for 1 h, then incubated with primary and secondary antibodies and visualized using a Clarity Western ECL kit (BIO-RAD, Hercules, CA, USA). The membranes were stripped using Restore Western blot to blot with a loading control antibody.

## 4. Conclusions

Therapeutic targeting of the EphA2-LBD has been pursued in recent years by a variety of approaches [27,32,37,38,39,40,41,42,43,44,45,46,47,48]. Here, using a combination of biophysical and cellular assays we conclude that dimeric 12mer agonistic agents can induce EphA2 receptor dimerization and subsequent activation and degradation, compatible with a proposed molecular model on ligand-induced EphA2 dimer formation. Recent observations reported an enhanced cell migration in EphA2 transfected cells, and such enhancement was even more pronounced when cells were transfected with EphA2 mutants that presented defective dimerization properties [35]. These data suggested that even transient dimerization of the receptor could have a profound effect in attenuating EphA2 driven cell migration [35]. Accordingly, we found that EphA2-KO BxPC3 pancreatic cancer cells presented reduced migratory properties that are comparable to WT-cells treated with either ephrinA1-Fc or our dimeric agonistic agents. We also noted that our agents induced a sustained degradation of the receptor over time similar to what we have observed with ephrinA1-Fc treatment. These data suggest that 135H12, or perhaps further and even more potent similar dimeric compounds, could be used in combination therapies in an attempt to suppress pro-oncogenic EphA2 signaling. In addition, similar to what we have recently proposed [24,25,26,27,28,29], we also envision derivatizing 135H12 or related agents with cytotoxic or imaging reagents, for targeted delivery of chemotherapy, or diagnostic purposes, respectively.

## Figures and Tables

**Table 1:** Sequences of 12-mers and their respective dimers cited in the manuscript. IC_50_ values (μM) were derived from the dissociation-enhanced lanthanide fluorescent immunoassay (DELFIA) assay. Reported standard errors represent number of experiments in with each having duplicate measurements. Hyp = trans 4-hydroxy-l-proline; Nle = l-norleucine; Hsr = l-homoserine; *GABA* = γ-aminobutyric acid.

ID	Sequence	IC_50_ (nM) (DELFIA)
123B9	(4F,3ClPhOCH_2_CO)SAYPDSVP(Nle)(Hsr)S-CONH_2_	6500 ± 1700, n = 2
YSA	H_2_N-YSAYPDSVPMMS-CONH_2_	16200 ± 800, n = 14
135B12	H_2_N-YSAYPDSVPFRP-CONH_2_	1600 ± 200, n = 16
135C11 (dimer of 135B12)	(H_2_N-YSAYPDSVPFRPG)_2_-K-CONH_2_	700 ± 100, n = 4
135C12 (dimer of 135B12)	(H_2_N-YSAYPDSVPFRP-βAla)_2_-K-CONH_2_	300 ± 100, n = 3
135D1 (dimer of 135B12)	(H_2_N-YSAYPDSVPFRP-GABA)_2_-K-CONH_2_	400 ± 200, n = 3
135E2	(4F,3Cl-PhOCH_2_CO)SAYPDSVPFRP-CONH_2_	3100 ± 600, n = 3
135G3	(4F,3Cl-PhOCH_2_CO)SAYPDSV(Hyp)(4Cl-Phe)RP-CONH_2_	600 ± 100, n = 6
135G4 (dimer of 135G3)	((4F,3Cl-PhOCH_2_CO)SAYPDSV(Hyp)(4Cl-Phe)RPG)_2_-K-CONH_2_	130 ± 2 n = 1
135H11	(3-CH_3_,6,7-OCH_3_,Benzofuranoic acid)LA(4-CH_3_-Tyr)PDA V(Hyp)(4Cl-Phe)RP-CONH_2_	130 ± 1, n = 4
135H12 (dimer of 135H11)	((3-CH_3_,6,7-OCH_3_,Benzofuranoic acid)LA(4-CH_3_-Tyr)PDAV(Hyp)(4Cl-Phe) RPG)_2_-K-CONH_2_	150 ± 60, n = 3
135I1 (tetramer of 135H11)	{[(3-CH_3_,6,7-OCH_3_,Benzofuranoic acid)LA(4-CH_3_-Tyr)PDAV(Hyp)(4Cl-Phe)RPG]_2_-K-K}_2_-K-CONH_2_	60 ± 10, n = 2
